# Biomechanical Behaviors and Degradation Properties of Multilayered Polymer Scaffolds: The Phase Space Method for Bile Duct Design and Bioengineering

**DOI:** 10.3390/biomedicines11030745

**Published:** 2023-03-01

**Authors:** Ilya Klabukov, Timur Tenchurin, Alexey Shepelev, Denis Baranovskii, Vissarion Mamagulashvili, Tatiana Dyuzheva, Olga Krasilnikova, Maksim Balyasin, Alexey Lyundup, Mikhail Krasheninnikov, Yana Sulina, Vitaly Gomzyak, Sergey Krasheninnikov, Alexander Buzin, Georgiy Zayratyants, Anna Yakimova, Anna Demchenko, Sergey Ivanov, Peter Shegay, Andrey Kaprin, Sergei Chvalun

**Affiliations:** 1 Department of Regenerative Medicine, National Medical Research Radiological Centre of the Ministry of Health of the Russian Federation, 249031 Obninsk, Russia; 2 Department of Urology and Operative Nephrology, Peoples Friendship University of Russia (RUDN University), 117198 Moscow, Russia; 3 Obninsk Institute for Nuclear Power Engineering, National Research Nuclear University MEPhI, 115409 Obninsk, Russia; 4 National Research Centre “Kurchatov Institute”, 1, Akademika Kurchatova pl., 123182 Moscow, Russia; 5 Department of Hospital Surgery, Sklifosovsky Institute of Clinical Medicine, Sechenov First Moscow State Medical University (Sechenov University), 119435 Moscow, Russia; 6 Research and Educational Resource Center for Cellular Technologies, Peoples Friendship University of Russia (RUDN University), 117198 Moscow, Russia; 7 N.P. Bochkov Research Centre for Medical Genetics, 115478 Moscow, Russia; 8 Lomonosov Institute of Fine Chemical Technologies, Russian Technological University MIREA, 119454 Moscow, Russia; 9 Department of Obstetrics and Gynecology, Sechenov First Moscow State Medical University (Sechenov University), 119435 Moscow, Russia; 10 Laboratory of the Structure of Polymer Materials, Enikolopov Institute of Synthetic Polymer Materials RAS, 117393 Moscow, Russia; 11 Department of Pathology, Moscow State University of Medicine and Dentistry, Delegatskaya st., 20, p. 1, 127473 Moscow, Russia

**Keywords:** bile duct, bioactive, biobased, biocompatibility, biomedical, copolymers, degradation, design, electrospinning, multilayered, multi-layered, physiological relevance, polycaprolactone, polyesters, regenerative medicine, scaffold, tissue engineering

## Abstract

This article reports the electrospinning technique for the manufacturing of multilayered scaffolds for bile duct tissue engineering based on an inner layer of polycaprolactone (PCL) and an outer layer either of a copolymer of D,L-lactide and glycolide (PLGA) or a copolymer of L-lactide and ε-caprolactone (PLCL). A study of the degradation properties of separate polymers showed that flat PCL samples exhibited the highest resistance to hydrolysis in comparison with PLGA and PLCL. Irrespective of the liquid-phase nature, no significant mass loss of PCL samples was found in 140 days of incubation. The PLCL- and PLGA-based flat samples were more prone to hydrolysis within the same period of time, which was confirmed by the increased loss of mass and a significant reduction of weight-average molecular mass. The study of the mechanical properties of developed multi-layered tubular scaffolds revealed that their strength in the longitudinal and transverse directions was comparable with the values measured for a decellularized bile duct. The strength of three-layered scaffolds declined significantly because of the active degradation of the outer layer made of PLGA. The strength of scaffolds with the PLCL outer layer deteriorated much less with time, both in the axial (*p*-value = 0.0016) and radial (*p*-value = 0.0022) directions. A novel method for assessment of the physiological relevance of synthetic scaffolds was developed and named the phase space approach for assessment of physiological relevance. Two-dimensional phase space (elongation modulus and tensile strength) was used for the assessment and visualization of the physiological relevance of scaffolds for bile duct bioengineering. In conclusion, the design of scaffolds for the creation of physiologically relevant tissue-engineered bile ducts should be based not only on biodegradation properties but also on the biomechanical time-related behavior of various compositions of polymers and copolymers.

## 1. Introduction

Recovery of bile flow in the case of bile duct damage and biliodigestive anastomosis stricture still remains among the key challenges in modern medicine [1,2,3]. The use of balloon dilatation and plastic stent installation is not efficient enough because of their frequent migration from the bile duct lumen, the need for re-intervention every three months due to stent lumen obturation by the bile salts, and potential complications that may occur during their repeated placement [4,5].

The interest in the use of biocompatible and biodegradable materials for biliary stent construction is motivated by their degradability in natural conditions and incorporation into the lumen wall. Hence, biocompatible and biodegradable materials can be used for the formation of tissue-engineered grafts that may represent an alternative to stents and help to avoid the risk of stent migration [3,6,7].

The principal condition for the successful use of a biodegradable scaffold is the retention of its functional properties, e.g., its strength and desirable biodegradation, for the required period of its placement. The prosthetic structure must be impermeable to bile during the whole replacement period. The degradation time, over which the prosthesis retains its functional properties, must be sufficient for scaffold material replacement with the native tissue of the patient [8].

To ensure the integrity of the bile-excreting functions, engineered scaffolds must match their natural equivalents in mechanical properties. The bile duct, like most other native tissues, demonstrates profound anisotropy in mechanical properties in the radial and axial directions, associated with the collagenous scaffold nature [9,10,11]. Certainly, this feature must be taken into account when manufacturing synthetic scaffolds.

Extensive studies, both in vitro and in vivo, determined the properties of various materials. The materials used were mainly polydioxanone [12] and copolymers of lactic acid [13,14,15,16,17]. The duration of the degradation process varies from 8 weeks to 13 weeks for polydioxanone [12] and from 6 months to 18 months for the mixture of L- and D-forms of polylactide [14]. At the same time, the optimal duration of stenting with standard plastic and metal biliary stents to achieve a positive result in patients with bile duct stricture is still inconclusive; the period reported was from 3 to 9 months [17,18,19].

In the first attempts at bile duct bioengineering, rubber and teflon were used [20,21,22]; however, long-term follow-ups showed the absence of biological compatibility of these materials. The use of polyesters opened a new era of biodegradable plastics in biliary surgery, including copolymers and composites. Advanced experimental studies of drug-resealing materials and cell-seeded tissue-engineered constructs uncovered the importance of the physiological relevance of material properties. These properties should both maintain the mechanical parameters of the native bile duct and its degradation rate to ensure the effective replacement of an implant with native tissue [22]. Knowledge of material degradation characteristics is indispensable for the creation of a tissue-engineered bile duct.

Biodegradable polymers such as polycaprolactone (PCL), poly-D,L-lactide-co-glycolide (PLGA), as well as L-lactide-co-ε-caprolactone (PLCL) are among promising candidates for bile duct tissue engineering. Using various fabrication modalities, it is possible to obtain PCL with different biodegradation behavior, porosity, hydrophilicity, and surface roughness [23]. PCL is presented in different molecular weights, allowing customization of its degradation rate and Young’s modulus [24]. PCL’s mechanical properties may also be adjusted by using certain methods of fabrication and by choosing the porosity level. For example, the tensile strength of porous and fibrous PCL scaffolds is lower than that of bulk PCL [25]. PCL-based constructs have been tested for bone and cartilage repair, enhancement of vascularization, dentin and pulp tissue regeneration, skin wound healing, heart valve tissue engineering, etc. [26,27,28,29,30].

The mechanical properties of fibrous PLGA and PLCL allow the formation of biocompatible and fast-bioresorbable scaffolds with a Young’s modulus of 17–22 MPa (PLCL) and 40–55 MPa (PLGA) [31,32,33,34,35,36,37]. The key challenge in the use of natural and polymeric materials is the difficulty in selecting the optimal balance between the biodegradation rate, mechanical stability, and immune response [38].

It is well known that the inadequate mechanical properties of implanted scaffolds may affect the viability of both cells seeded on the scaffold and cells in affected tissues adjacent to the scaffold. Increased stiffness of the scaffold compared to the surrounding soft tissues leads to the development of inflammation in the affected tissues and capsule formation. Insufficient scaffold’s stiffness stimulates the death of seeded or migrating cells and leads to the formation of a fibrosis or necrosis zone [39,40]. Achieving suitable mechanical properties of the implant requires finding the balance in properties of the material within a certain range.

Cell seeding does not lead to completely improved physiological relevance because of possible side effects, adverse events, and cell-associated complications [41,42]. Moreover, the migration of cells to regions with different degrees of stiffness may be an essential factor for keeping cells far from tensional equilibrium [43]. However, there are no standard models for the assessment of the physiological compatibility of materials based on their biomechanical properties. The physiological relevance of materials is essential for the formation of scaffolds for bioengineered organs. Currently, the design of tissue-engineered grafts for the bile duct that meets clinical requirements remains a grand challenge in bioengineering [3,44,45,46,47], because it requires a deep understanding of the material behaviors after implantation [48,49,50]. The electrospinning technique is one advanced method that allows the creation of microfibrous scaffolds with desired properties from various materials for use in tissue engineering, treatment of infected wounds, etc. [51,52], and it may be used for the controlled formation of multilayered scaffolds.

Previously, our study showed that proliferation of epithelial cells on manufactured scaffolds was dependent on the method of their modification or functionalization [53]. We showed that synthetic scaffolds based on linear polyesters retained 30–50% of their initial strength after 3 months of exposure in a model media simulating the environment of bile ducts [54]. We hypothesized that a multi-layered design would be beneficial for the creation of a bioengineered equivalent of the bile duct based on polymers with various degradation properties (PCL, PLCL, and PLGA) using an electrospinning technique [55,56,57]. 

This study aims to explore the creation of a multilayered bile duct scaffold with mechanical properties similar to those of a native bile duct and to perform a comparative analysis of variation in the molecular structure of PCL, PLCL, and PLGA during their degradation in different media and time conditions.

## 2. Materials and Methods

### 2.1. Polymeric Materials

To study degradation kinetics, we used flat scaffolds fabricated from biocompatible polymers using the electrospinning method. The polymers investigated were as follows: polycaprolactone (PCL: M_w_ = 138 kDa, M_w_/M_n_ = 1.2, Sigma Aldrich), 70:30 copolymer of D- and L-lactide with glycolide (PLGA: M_w_ = 104 kDa, M_w_/M_n_ = 1.7, synthesized in NBICS Center of the Kurchatov Institute), and 70:30 copolymer of L-lactide with ε-caprolactone (PLCL: M_w_ = 137 kDa, M_w_/M_n_ = 1.5, Corbion Purac). The spinning solutions were prepared with chemically pure-grade solvents: chloroform (Komponent-Reaktiv, LLC) and ethanol (FarmReserv, LLC).

The relative lactide:ε-caprolactone composition was studied with Nuclear Magnetic Resonance (1H-NMR) using signals of CH-group protons of lactide and ε-CH2-group protons of ε-caprolactone.

### 2.2. Fabrication of Flat Scaffold Samples

The flat fibrous scaffolds were fabricated from PCL, PLCL, and PLGA solutions in a 9:1 mixture of chloroform and ethanol. For the preparation of the scaffolds from nonwoven fibrous materials, the electrospinning process variables were selected to ensure the fibers were spinning at room temperature and that the relative humidity was maintained at 20% to 30%. The detailed process conditions are listed in Table 1. A grounded metal cylinder with a diameter of 70 mm rotating at a low speed of 20 rpm was used as a collecting electrode. 

### 2.3. Electrospinning of Tubular Multi-Layered Scaffolds

**Figure 1:**
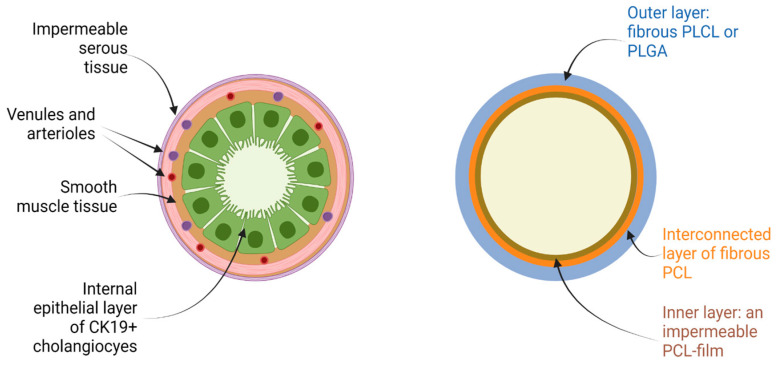
Structure of the native bile duct and multilayered design of the bile duct scaffolds used in the present study. Figure was produced using BioRender platform.

The tubular scaffolds were fabricated by electrospinning using a rotating metal cylinder (20 rpm) with a diameter of 4.3 mm. After the formation of the inner layer of nonwoven fibrous PCL, the inter-electrode gap was changed to allow the application of a wet PCL fibrous interconnected layer. The thickness of the layer at the beginning exceeded 0.01 mm. To form the outer layer, the fibrous layers of either PLGA or PLCL were deposited on the interconnected PCL layer (Figure 1). 

The tubular multi-layered scaffold samples were fabricated with the same concentration of polymer solutions and spinning parameters that were used for the fabrication of flat samples. All fabricated samples were dried in a vacuum of 1 mbar for 24 h in order to completely remove the solvent. The samples were packaged in CLINIPAK pouches and subjected to sterilization with a 15 kGy dose of gamma radiation.

### 2.4. Degradation of Flat Polymeric Scaffolds

Before the start of the experiment, nonwoven fibrous materials were degassed under reduced pressure to improve their wetting ability. The degradation kinetics of the flat polymeric scaffolds were studied in various media: distilled water (water), phosphate buffer saline (PBS), Fenton’s reagent (FR), culture medium with fetal bovine serum (DMEM + 10% FBS) and without it (DMEM), and ox bile (bile). The extent of scaffold degradation was assessed by investigating changes in the specimens’ mass, mechanical properties, and molecular mass. The destruction studies were carried out with an incubation period of 14 days or more at a temperature of 37 °C. The further incubation interval of the samples was determined according to the test result. 

After removal from the media, each specimen was immersed in distilled water to eliminate low-molecular-weight substances. The samples were placed in a 200 mL cone flask containing 100 mL of water, and the flask was installed in the laboratory shaker PE-6410 (Ecros, Saint Petersburg, Russia) for 1 h. This operation was repeated three times. Afterward, the samples were dried to a constant weight in the vacuum oven. To determine mass loss, the samples were weighed before and after the experiment.

The molecular characteristics of the samples after degradation were determined by gel permeation chromatography (GPC) using the Knauer chromatography system with a refractive index detector and a Phenogel column, 300 mm × 7.8 mm, with a particle pore size of 1 µm. The columns were calibrated by polystyrene standards. The tests were carried out at 40 °C with an eluent flow of 1 mL per minute. The polymer samples were dissolved in tetrahydrofuran at a concentration of 2–5 mg per mL. Before injection into the chromatograph, the solutions were filtered through the syringe filter with a hydrophobic polytetrafluoroethylene membrane with 0.45 µm pores.

### 2.5. Decellularization of a Native Bile Duct

An enzymatic-detergent decellularization protocol was used for the processing of human native bile duct specimens. The post-mortem usage of human tissue was approved by the Local Ethics Committee of Sechenov University (protocol code #03-17, date of approval, 19 April 2017).

Bile duct specimens were pre-washed in PBS and placed in an ice-cold, sterile aqueous DMSO solution (6 vol.% Sigma-Aldrich, St. Louis, MO, USA) with 1% of penicillin, streptomycin, and fungizone for 24 h. Specimens were rinsed in distilled water and underwent a 4-h incubation in an ice-cold sodium deoxycholate (4 vol.% Sigma-Aldrich, USA). After that and prior to enzymatic treatment, the samples were left in Hanks’ balanced salt solution for 0.5 h. Next, isolated bile duct specimens were treated with nuclease (2 U/mL DNase I solution in 1 M/L NaCl, all from Sigma-Aldrich, St. Louis, MO, USA) combined with DMSO (10 vol.%). Then, the patches were exposed to detergent substances in a solution containing Triton X-100 (1 vol.%), CHAPS (0.25 vol.% with 50 mM tris-HCl, pH 8.0, all from Sigma-Aldrich, St. Louis, MO, USA), and DMSO (10 vol.%) for 48 h. In order to avoid any residual detergent amount in the tissue, the following PBS-washing step was prolonged to 48 h with regular changing of the solution (every 12 h). Incubation in a sodium deoxycholate and nuclease solution was then repeated in order to provide sufficient DNA elimination. Finally, the patches were rinsed 3 times for 24 h in PBS and stored at −80 °C until analyzed. All steps were carried out with the use of ice-cold solutions, with the exception of the DNase solution, which was preheated to 37 °C.

### 2.6. Mechanical Properties of Native and Decellularized Bile Duct and Synthetic Tubular Scaffolds

**Figure 2:**
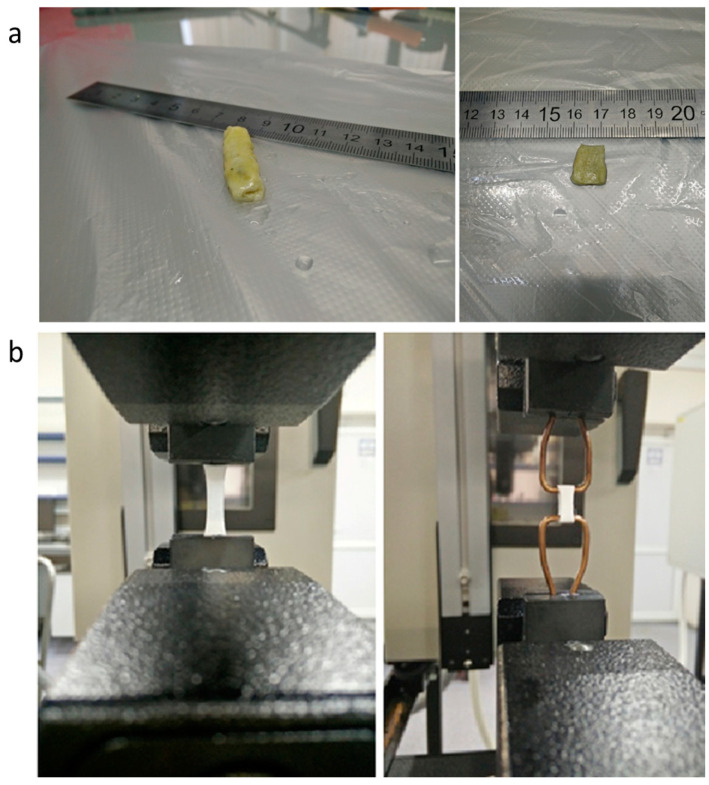
(**a**) Image of a decellularized bile duct specimen before the testing in various test modes; (**b**) Synthetic scaffolds of bile ducts in various modes of testing.

Mechanical properties of native and decellularized bile ducts and synthetic scaffolds were determined by the Instron-5965 Universal Testing System (Thermo Fisher Scientific, Waltham, MA, USA). The rate of deformation was 10 mm per min. The strength of native and decellularized bile ducts in the axial direction was measured using rectangle-shaped specimens with a test portion size of 10 mm × 10 mm, while synthetic specimens had a size of 10 mm × 5 mm. For mechanical tests in the radial direction, the ring-shaped, 5-mm-wide specimens were used. Prior to testing all specimens of native and decellularized bile ducts, the specimens were immersed in phosphate buffer saline. All synthetic scaffolds were immersed in the same medium. The conditioning time at 23 °C was no less than 4 h. Decellularized specimens of the bile duct are shown in Figure 2a. 

The technique of mechanical testing is shown in Figure 2b. Specific mechanical properties were calculated using the linear dimensions measured. Specimen thickness was determined as the mean arithmetic value of three points located no less than 5 mm from each other. The strength of synthetic scaffolds was calculated using the nominal cross-section based on the specimen mass and density of the corresponding polymer. The elastic modulus was determined from the maximum slope of the stress-strain curve. Mechanical properties are presented with the standard deviation. 

### 2.7. Scanning Electron Microscopy of Flat Polymeric Scaffolds

Specimen surface morphology was studied with the scanning electron microscope Scios (FEI Company, Hillsboro, OR, USA) in a high vacuum (<10–4 Pa). The images were obtained with the Everhart–Thornley detector of secondary electrons (ETD) at an ultra-low accelerating voltage of 1 kV. These experimental conditions have enhanced the topographic contrast on the fiber’s surface, significantly reduced the charge accumulation, and improved the spatial resolution. The analysis of fiber dimensions as well as size distribution was conducted with Scope Photo Image Software (ScopeTek, Hangzou, China). To calculate the average diameter of the fiber, measurements of no fewer than 100 fibers were performed. 

### 2.8. Differential Scanning Calorimetry

The thermophysical behavior of specimens was studied with the Perkin-Elmer DSC8500 (PerkinElmer, Waltham, MA, USA) differential scanning calorimeter (DSC). The DSC cell was flushed with dry nitrogen at a flow rate of 20 mL per minute. An IntraCooler III mechanical refrigerator was used for cooling. The specimens were weighed on a Sartorius microbalance with an accuracy of ±0.01 mg. The heating rate was 20 °C per min.

### 2.9. Cytocompatibility Evaluation

Sample cytocompatibility was tested by MTT assay with the use of 3T3 and MCF-7 cell cultures. In a 96-well plate, cells were seeded on circular samples of flat polymeric scaffolds with diameter 0.6 mm at a cell density of 4200 cells/well (for 3T3 cells) and 7200 cells/well (for MCF-7 cells). On day 7, the MTT assay was performed according to the standard protocol [6]. The optical density (OD) was measured on a Multiskan FC spectrophotometer (Thermo Fisher Scientific, Waltham, MA, USA) at a wavelength of 540 nm. The results showed relative biocompatibility of materials as = (OD of cells seeded on the material − OD blank)/(OD of cells on the Positive control well − OD of cells on the bottom of the well with scaffold).

### 2.10. Statistics

Statistical data processing was performed using variance statistics with the GraphPad Prism 7 computer program (GraphPad Software Inc., LaJolla, CA, USA). Statistical comparisons were made by a one-way and two-way analysis of variance (ANOVA). A post-hoc Tukey test was used to evaluate differences among groups. A *p*-value < 0.05 was considered statistically significant.

## 3. Results

### 3.1. Mechanical Properties of Native and Decellularized Bile Duct

Native and decellularized samples, as well as tubular samples of an artificial scaffold, were subjected to tests in the radial and axial directions (Table 2). All tensile curves had a distinct S-shape, and the elastic modulus was determined from the maximum slope.

Decellularized samples exhibited a higher strength and elastic modulus, preserving the high deformability, perhaps due to the increased sample density after cellular component extraction and, as a consequence, enhanced interaction between individual fibrils of the elastin-collagenous scaffold. This finding also indirectly indicates that the elastin-collagenous scaffold remains intact in the decellularization process.

### 3.2. Mass Loss of Flat Polymeric Scaffolds during Degradation

The measurement of a synthetic scaffold mass loss during degradation is quite a difficult task due to the highly dispersed state of the material. Nevertheless, it was established that PCL-based flat scaffolds do not lose mass substantially over 140 days. The mass loss during the degradation of PCL-based scaffolds in aqueous medium was very low, despite the higher specific surface [58,59,60]. In PLCL-based scaffolds, the mass loss reached 10–14% over 70 days, and after 140 days, the specimens were destroyed completely. The scaffolds derived from PLGA were found to be the least stable. After 14 days, it was very difficult to measure the mass, and afterward, the specimen crumbled.

### 3.3. Changes in Fibrous Morphology of Flat Scaffolds during Degradation 

The changes in the surface morphology of fibrous biocompatible scaffolds at the initial stage of degradation were studied by scanning electron microscopy (Figures S1–S3). The original scaffolds of PCL, PLCL, and PLGA have a relatively broad diameter distribution that appears to be a result of the splitting of the jet in the electric field. The “beads” discovered in the fibrous structure of PCL-based specimens were the result of a weak intermolecular network in the spinning solution. The stability of nonwoven PCL-based materials was confirmed by comparing micrographs of specimens after their removal from all test media with the original ones (Figure S1). No significant morphological changes could be found in all obtained images. The micrographs of nonwoven materials derived from PLCL and PLGA showed pronounced partial destruction of fibers after 14 days (Figures S2 and S3). 

The fiber cracks are indicated in the micrographs by arrows. It was discovered that the changes in the fiber diameter distribution were the most pronounced after degradation in bile and H_2_O_2_ (Figures S2 and S3). An increase in the average diameter of PLGA fibers was also observed [61]. Such structural transformations are most likely observed because the most active destruction occurs in the thinnest fibers due to their higher specific surface. It is worth noting that there was a notable shrinkage of PLGA-based specimens after incubation in all kinds of test media. On average, the surface area of the scaffolds studied shrank by 32%. The surface area of specimens prepared from other polymers remained unchanged.

### 3.4. Variation of Molecular Mass Characteristics of Flat Polymeric Scaffolds

**Figure 3:**
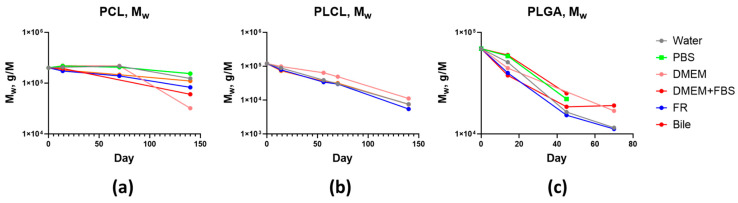
Decrease in the molecular mass of scaffolds based on: (**a**) PCL; (**b**) PLCL; (**c**) PLGA.

**Figure 4:**
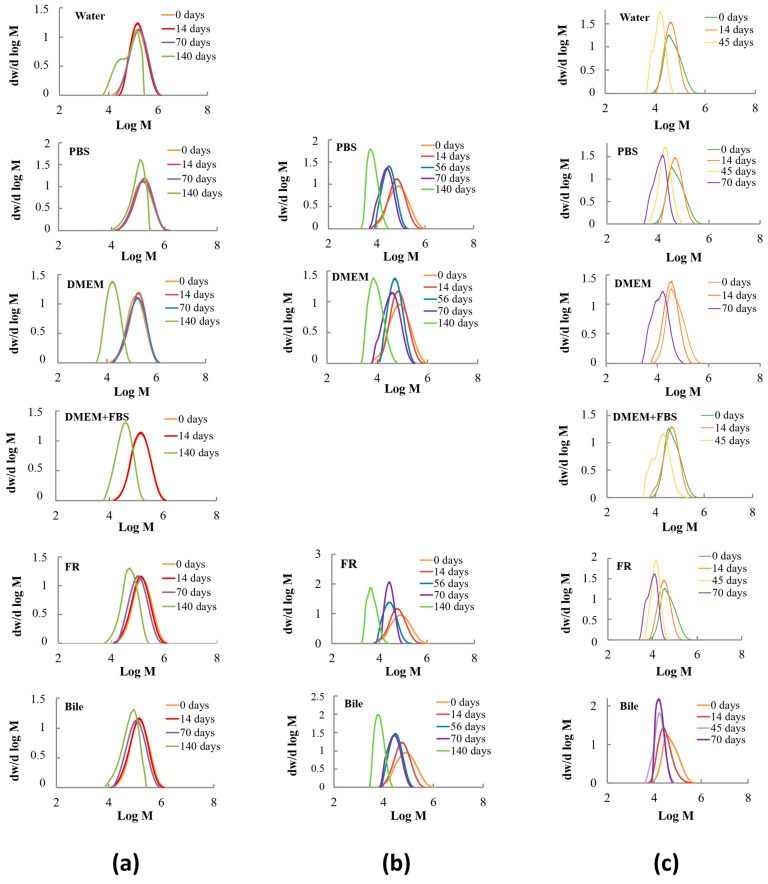
Variation of molecular mass distribution in biocompatible scaffolds derived from: (**a**) PCL; (**b**) PLCL; (**c**) PLGA depending on test media composition and degradation time.

The initially sterilized samples of fibrous materials were used to compare the values of weight-average molecular weight (M_w_) and polydispersity index (M_w_/M_n_). The changes in molecular characteristics of flat polymeric scaffolds depending on time and conditions of degradation are presented in Figure 3 and Figure 4. 

It is evident that molecular mass loss is accelerated with time. The self-catalyzed character of destruction is determined by the accumulation of acid released during the degradation of polyester bonds. Let us consider the effect of the test medium on the decrease of weight-average molecular mass as a function of time. After 14 days, the chain scission was most pronounced in the bile and FR media. The molecular masses of PCL, PLCL, and PLGA in the phosphate buffer saline and water decreased much less. After 45 days, the molecular mass of PLGA dropped below 26 kDa, and the scaffolds started to disintegrate. At this degradation stage, the difference between the catalytic activities of the test media disappears. Similar behavior was observed during the destruction of PLCL-based scaffolds [62]. Approximately, the same level of PLCL molecular mass decrease occurred in bile, FR, and phosphate buffer saline. A lesser reduction of the molecular mass of PLCL was found in DMEM. A similar level of PLCL molecular mass decrease was found in different media and was observed for up to 140 days. When the molecular mass dropped below 12 kDa, the PLCL-based scaffolds completely lost their mechanical properties. A remarkable difference was observed during the degradation of PCL. After 140 days, the molecular weight of PCL in the culture medium decreased to 24–45 kDa, which is much greater compared to bile and FR (62–82 kDa). The catalytic activity of water and especially of the phosphate buffer saline was even lower.

In the course of hydrolysis, the peak of the molecular mass distribution of PCL, PLCL, and PLGA shifted to a lower molecular mass (Figure 4) and often revealed unimodal behavior. In some cases, a poorly defined oligomeric fraction appeared at the final stage of degradation together with the main peak. The bimodal distribution can be associated with the different rates of ester group degradation in the bulk and on the surface of the material, or in the amorphous and crystalline portions of the polymer [63,64]. GPC data demonstrated that the destruction rate increased in the following sequence: PCL, PLCL, and PLGA. The micrographs of materials removed from the test media after 14 days (Figures S1–S3) confirmed GPC results. Furthermore, homopolymers degrade slower compared to copolymers [65,66,67]. 

### 3.5. Changes in the Mechanical Properties of Flat Scaffolds during Degradation

**Figure 5:**
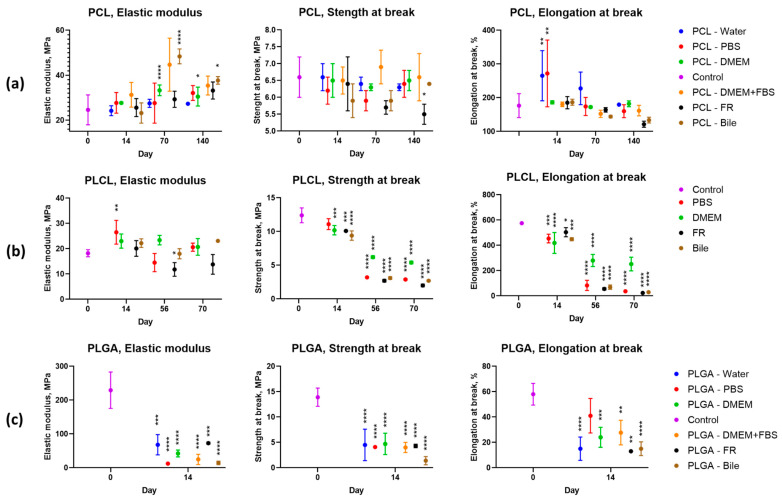
Dependence of elastic modulus, strength, and strain at break of biocompatible scaffolds based on: (**a**) PCL; (**b**) PLCL; (**c**) PLGA on conditions of degradation process. Control—mechanical properties of original samples. *—*p*-value < 0.05; **—*p*-value < 0.01; ***—*p*-value < 0.001; ****—*p*-value < 0.0001.

Let us consider the changes in elastic modulus, strength, and relative elongation during the degradation of the polymers studied. Figure 5 presents the results of the study of the mechanical properties of biocompatible scaffolds as a function of the test medium and degradation time.

The elastic modulus of PCL increases slightly with time in all test media. The elastic modulus of PLCL incubated in phosphate buffer saline, cell medium, and bile did not change substantially in comparison to the original value throughout the test run. In the FR, the value decreased by 35–45% after 70 days.

The elastic modulus of PLGA-based scaffolds dropped to an extremely low value after 14 days of incubation in all test media.

The strength of the PCL scaffolds remained unchanged during the whole experiment in all media with the exception of FR, where a gradual decrease in the strength properties of the samples was observed. 

The strength of PLCL-based scaffolds deteriorated gradually. At the initial stage, the strength decreased much faster in the FR, phosphate buffer saline, and bile. In the PLGA scaffolds, the strength drops down after 14 days.

The relative elongation of PCL samples after 14 days of incubation in water and phosphate buffer saline increased significantly, but with a broad scatter of experimental data. Further incubation in water, phosphate buffer saline, and DMEM cell culture medium with or without serum did not lead to substantial changes when compared to the control sample. In the media containing more aggressive substances (FR and bile), the relative elongation decreased with time.

The rate of degradation of scaffolds in aqueous media depends significantly on the degree of crystallinity. The data indicate that the nonwoven PLGA material is amorphous. During heating, devitrification at 50 °C with an endothermic peak due to enthalpy relaxation is observed (Figure S4). In PLCL-based samples, the lactide is amorphous. Its glass transition is observed at 25 °C. Such a low glass transition temperature is associated, most probably with its plastification by a caprolactone component of the copolymer. In nonwoven materials based on PCL and PLCL, the caprolactone is partially crystalline. In the first case, the polycaprolactone crystallinity is about 35%, while in the second case, it is only 5% (Figure S4B,C). The caprolactone crystallization in the copolymer suggests the existence of a micro-block chain structure. The absence of a polylactide crystalline phase can be explained by quenching in the electrospinning process due to the fast cooling of the polymer jet. The higher degree of crystallinity in PLC-based materials explains the longer time for these scaffolds to degrade because the hydrolysis affects the less dense amorphous regions, which are easily accessible by water. For this reason, the crystallinity of the PCL material can increase in the course of hydrolysis [68,69] which can be a possible reason why the elastic modulus of PCL-based materials increases with time.

The thermophysical methods were applied to study the flat biocompatible scaffold degradation as a function of their supramolecular structure. The DSC curves from the first heating of the samples are presented in Figure S4.

### 3.6. Cytocompatibility Evaluation

**Figure 6:**
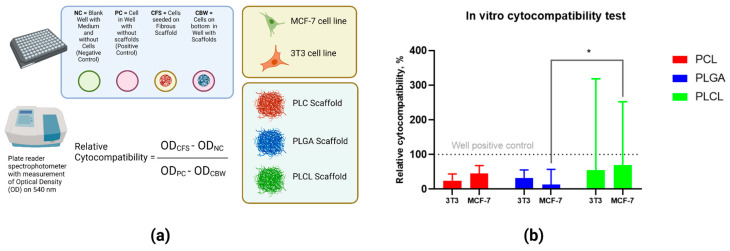
(**a**) Technique for the assessment of cytocompatibility of polymeric materials by seeding with MCF-7 and 3T3 cells, results presented on day 7; (**b**) Results of the MTT assay of PCL, PLGA, and PLCL. *—*p*-value < 0.05.

PLCL was more cytocompatible with both cell lines in comparison with PCL and PLGA, but these differences were not statistically significant (Figure 6). The two-way ANOVA assay did not show any statistical differences between scaffolds, excluding PLCL and PLGA, when seeded with MCF-7 cells (*p* = 0.021).

### 3.7. Biomechanical Behavior of Tubular Synthetic Scaffolds

To ensure bile excretion, the scaffolds must resist the bile attack for the required period of time and withstand the longitudinal and transverse forces that develop during scaffold functioning. To study the biomechanical behavior, the tubular scaffolds with different compositions (PCL + PCL + PLGA or PCL + PCL + PLCL) were incubated in bile for 14 and 70 days. 

Visual examination of the samples removed from the bile revealed that the surface layer of PLGA tubular scaffolds was partially preserved after 14 days of bile exposure. After 70 days, the surface layer had decomposed almost completely. Whereas, the surface layer of PLCL scaffolds was preserved quite well during the full length of the experiment.

**Figure 7:**
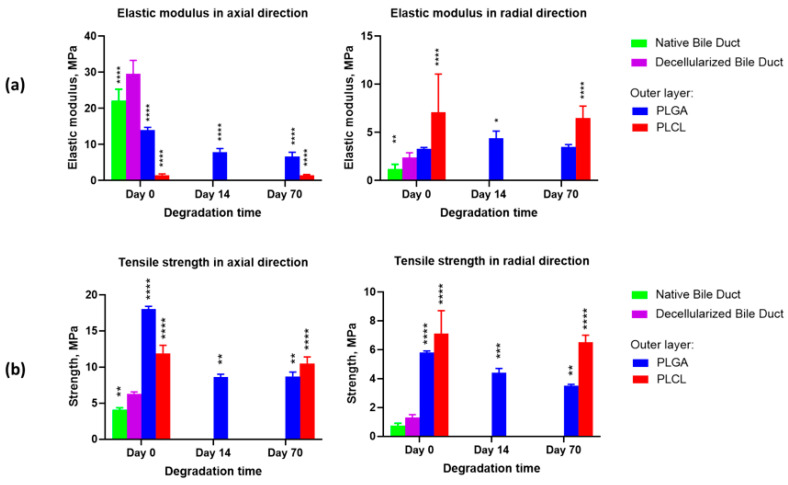
The results of the mechanical properties investigation in the tubular scaffolds with an outer layer derived from PLGA or PLCL, after degradation in bile: (**a**) Elastic modulus in the axial and radial directions; (**b**) Tensile strength in the axial and radial directions. Statistical significance is shown in comparison to the decellularized bile duct properties: *—*p* < 0.05; **—*p* < 0.01; ***—*p* < 0.001; ****—*p* < 0.0001.

From estimating the mechanical properties of fabricated scaffolds (Figure 7), it can be concluded that these characteristics fit the longitudinal (6.3 MPa) and transverse (1.3 MPa) strengths of the decellularized bile duct. 

It is worth noting that the results obtained are in agreement with the previously reported data on the mechanical properties of the native and decellularized tissues. For example, in the mitral valve of sheep (ovine), the strength increased from 1.23 MPa to 2.12 MPa [70], and in the mitral valve of pigs, from 11 MPa to 17 MPa [71]. Such an increase in tensile strength is explained by the change in the extracellular matrix pattern during decellularization and the absence of the cell component and ground substance tissues [71]. Figure 7 presents the results of the investigation of the tubular scaffolds’ elastic modulus and strength after their bile exposure for 14 and 70 days. The strength of the scaffolds declined significantly because of the active degradation of the outer layer prepared from PLGA. The strength of the scaffolds with the PLCL outer layer deteriorated much less with time, both in the axial (*p*-value = 0.0016) and radial (*p*-value = 0.0022) directions.

## 4. Discussion

The surgical implantation of the graft in the patient’s body causes an injury with the occurrence of an inflammatory and reparative reaction [72,73]. Then, the activated macrophages start to move to the injury site, penetrate into the structure of the biocompatible material, and release the oxidative degradation products (oxygen free radicals) [74,75]. The chemical bonds of macromolecules break down during oxidative degradation, resulting in a gradual decrease in the molecular weight of the polymer. In the hydrogen peroxide oxidizing medium, the process of polymeric chain destruction occurred mainly in a similar manner, allowing us to simulate the process of inflammation during the introduction of biomaterials into living organisms [76,77].

The longitudinal strength of the duct is more than twice as high as the transverse, which is in accordance with the data on the mechanical properties of animal tissues [78,79,80]. This ratio can be explained by the predominantly longitudinal direction of the fiber arrangement in the duct wall, along which the proliferation of epithelial cells is maximal [81,82].

The basic mechanism of biocompatible scaffold destruction (hydrolytic cleavage of the ester bonds) is a second-order reaction and can be catalyzed in strongly acidic (pH = 1–2) and alkaline media (pH = 13–14) [83,84], as well as in the presence of polyvalent acid anions [85]. The pH of biological liquids varies from 5 to 9, with a few exceptions [86,87]. Therefore, it is very important to study the kinetics of scaffold degradation as a function of the chemical composition of medium that is in contact with it in the human body, such as blood serum, extracellular fluid, and bile.

The extracellular fluid contains the ions Na^+^, Cl^−^, HCO_3_^−^, PO_4_^3−^, Mg^2+^, Ca^2+^, SO_4_^2−^, amino acids, proteins, and other substances. Consequently, DMEM and DMEM + 10% FBS media with similar chemical compositions, as well as PBS, were used to simulate the process of scaffold degradation to investigate the catalytic effect of phosphate ions [88].

The study of biocompatible scaffold hydrolysis in bile was of particular interest. The bile, with its high dispersing activity due to the presence of bile acids and salts, is capable of increasing the rate of the ester bonds’ cleavage, significantly [89,90].

The results of our study offer evidence that the process of degradation depends significantly on the media composition and chemical structure of the scaffolds. Among the studied media, bile and FR undoubtedly showed catalytic activity when compared to water and PBS. In the conditions of synchronized action of various media inside the body, the process of biomaterial degradation inside the body can be accelerated. In the case of PCL, the established catalytic activity was demonstrated by DMEM+10% FBS and DMEM medum. Presumably, it is associated with the small Fe^3+^-ions content in the culture media which increases the PCL hydrolysis rate [91,92].

Differential scanning calorimetry (DSC) was performed for the copolymer PLCL (70:30) and presented in Figure S4. Caprolactone in this composition crystallizes weakly, while lactide crystallizes much better. The microstructure of the copolymer was studied by nuclear magnetic resonance (NMR), and it was found that the average length of the lactide block is 3.6 units and that of the caprolactone block is 1.5 units. However, we believe that these are the average block lengths and that the microstructure may be on a gradient due to the different reactivity of lactide and caprolactone [93]. Thus, the copolymer is represented by chains enriched with lactide blocks at the beginning and long caprolactone blocks at the end. However, this gradient structure cannot be characterized on the finished polymer; but it is only possible to calculate the average block lengths. The relative PLCL composition was determined by 1H-NMR with the use of CH-group protons of lactide and ε-CH2-group protons of ε-caprolactone signals. Identification of spectra and microstructure calculation can be found in the paper (Morokov et al., 2020) [93].

Another factor contributing to the enhanced resistance to degradation of the caprolactone-based polymers is the higher number of methylene groups in their chains and the reduced availability of ester groups for hydrolysis [55,90,94,95]. These complementary factors reduce the ester group stability in the absence of side substituents and result in the autocatalytic action exerted by the higher concentration of carboxyl groups. The reported data indicate the enhancement of the biodegradability of polymer scaffolds in the following sequence: PCL, PLCL, and PLGA.

The polyester chains broke down in the course of hydrolysis, resulting in the subsequent deterioration of the mechanical properties and gradual mass loss of the scaffolds. The changes in molecular mass during degradation were studied by GPC. The presence of microorganisms strongly affects the rate of molecular mass reduction of polyesters. Consequently, the experiment was conducted in sterile conditions. Sterilization by gamma radiation can result in both the scission of existing molecular bonds and the formation of new ones in the polymer chains. It is clear from the review by Rediguieri et al. (2016) and from our findings that gamma radiation can affect the molecular mass of polymers, the tensile modulus, and the strength of the fibrous materials derived from them [96].

Until now, dependence on the degradation of polymeric materials on a specific surface area was not explored [97,98]. However, highly dispersed fibrous materials will have a higher rate of degradation. To determine the effect of the specific surface on the degradation process, we compared the existing literature on films and rods with the results obtained in this paper. As presented in Table 3, the process of degradation of polymer materials depends on the specific surface area. To better understand the results obtained, it is necessary to analyze the mechanism of degradation of polymeric materials. Initially, we can distinguish between two possible mechanisms for the decomposition of polymers. The first mechanism is associated with the occurrence of erosion, mainly in the surface layer of the polymer. The second mechanism relates to polymer swelling in liquid media, in which case erosion proceeds primarily in the implant volume. Due to the accumulation of terminal autocatalytic groups in the volume of the polymer, the kinetics of the degradation process are accelerated. The criterion for determining the mechanism in one or another case is the ratio between the rate of diffusion of water into the volume of the polymer and the rate of degradation of the ester bond [55,90,99,100,101,102,103].

Theoretically, PLGA, PLCL, and PCL may undergo bulk degradation [85,104,105]. Therefore, the autocatalytic effect should have a significant effect on the degradation process for these polymers. Indeed, as can be seen from Table 3, materials based on PLGA and PLCL with lower specific surfaces are degrading much faster. This degradation is due to a higher concentration of terminal autocatalytic groups in the bulk of the polymer. However, PCL has a high degree of crystallinity and hydrolytic stability in its ester bonds, resulting in the accumulation of terminal groups in the bulk of the polymer proceeding slowly; there is no experimental autocatalytic effect, which leads to long periods of degradation in the films and rods [106,107,108,109]. This structure causes a higher speed of degradation in high-dispersed fibrous PCL materials in comparison to films and rods. This structure may also be associated with an increased accessibility of ester bonds to water molecules and catalytically active substances in the surface layer and has a significant effect on the specific surface degradation [97,98,110].

The principal condition for the successful use of a biodegradable scaffold is that it retains its functional properties, e.g., its strength and desirable biodegradation period [111,112] for the required period of time. Tissue-engineered implant structures must provide impermeable walls to bile during the whole replacement period. The degradation time over which an implant retains its functional properties must be sufficient for scaffold material replacement with the native tissue of the patient.

The problem of physiological compatibility is that insufficient strength leads to traumatization of the implant, while excessive strength leads to traumatization of surrounding tissues. We developed a novel model for the assessment of the physiological compatibility of the implants. We chose two keystone parameters: elongation modulus (affects cells viability and robustness) and tensile strength (material robustness). Thus, the model of physiological relevance is based on the representation of the scaffold parameters as the two-dimensional phase space (elongation modulus and tensile strength).

**Figure 8:**
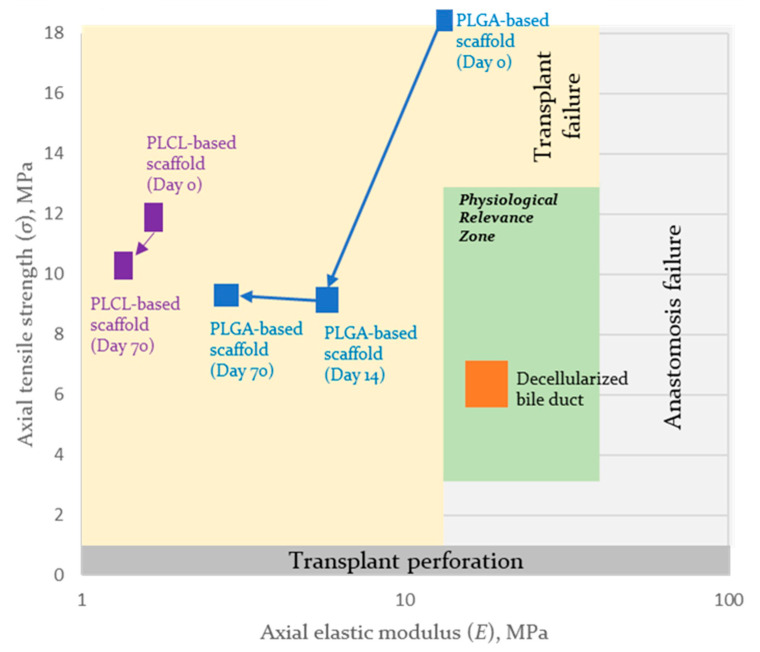
The zone of physiological relevance of the axial properties of the synthetic scaffolds for bile duct bioengineering in the phase space (elastic modulus and tensile strength) and trajectory for PLCL- and PLGA-based scaffolds during biodegradation after 14 and 70 days of exposure.

For bile duct scaffolds, the zone of biomechanical parameter stability was assessed as −30% and +100% of native decellularized bile duct parameters [113]. For example, the zone of physiological relevance of the scaffolds for bile duct bioengineering formed from three-layered scaffolds is presented in Figure 8.

In the schema (Figure 8), it is easy to see that the implantation of PLCL- and PLGA-based scaffolds may lead to graft failure due to their insufficient mechanical properties. At the same time, if implanted, the properties of the PLGA-based scaffold are close to those of the PLCL-based scaffold on day 70, due to the degradation of the PLGA layer. None of the mechanical properties of the formed scaffolds are within or cross the physiological relevance zone. This finding indicates possible ways to improve scaffolds by increasing the initial Young’s modulus, within which the obtained tensile strength should be retained.

## 5. Challenges and Perspectives

The lack of quantitative models for physiologically relevant tissue engineering hinders the design of artificial organs. Even the creation of anatomically simple subjects like hollow epithelial organs still represents a challenge during the planning stages of tissue growth and material degradation. 

Tissue engineering strategies that incorporate computational simulation of organ design may lead to more successful outcomes and more predictable clinical translation [48,49]. At the same time, the design of tissue-engineered constructs has tissue- and organ-specific features and requires the consideration of anatomical and functional properties [50]. Previously, an approach balancing the mechanical and biological compatibility of polymers was used for designing tissue-engineered vessels [55,114]. The principles applied to the creation and clinical application of biomedical products for regenerative medicine are still under development [115], but in recent years, an increasing volume of papers have been devoted to the problems of tissue engineering [116,117,118,119]. Indeed, the use of bioabsorbable bile duct substitutes for bile duct regeneration constitutes a promising approach that will be clinically useful in the future [119].

The design of smart scaffolds for the creation of tissue-engineered grafts requires not only new materials and methods for their modification [120], but also quantitative models for the assessment of their physiological relevance [121,122]. Therefore, the design of tissue-engineered grafts that fulfill clinical requirements remains a grand challenge for the creation of physiologically relevant tissue-engineered organs and tissues.

## 6. Conclusions

A three-layered bile duct scaffold can be formed on the basis of PCL with an outer layer either of PLCL or PLGA with the use of the electrospinning method. The scaffolds’ degradation process was studied as a function of their molecular composition and liquid-phase nature. The study showed the possibility of manufacturing PCL-based scaffolds with mechanical characteristics matching those of a decellularized bile duct. Thus, we can conclude that the design of scaffolds for tissue engineering may be based on the biodegradation and biomechanical time-related behaviors of various compositions of polymers and copolymers. To this date, the design of tissue-engineered grafts that completely correspond to clinical requirements remains challenging. The phase space approach (elongation modulus and tensile strength) for assessment and visualization of the physiological relevance of scaffolds for bile duct bioengineering was proposed to aid in the creation of physiologically relevant tissue-engineered bile duct.

## Figures and Tables

**Table 1:** Parameters of flat scaffold fabrication in the electric field.

No.	Polymer	Concentration (wt.%)	Volumetric Flow Rate of Solution, mL/h	Applied Voltage, kV	Inter-Electrode Gap, cm
1	PCL	7	8	14	25
2	PLCL	12	30	14	25
3	PLGA	14	30	14	20

**Table 2:** Mechanical characteristics of native and decellularized samples of bile duct.

Sample	Test Direction	N	σ, MPa	ε, %	*E*, MPa
Native	radial	3	0.75 ± 0.15	135 ± 14	1.2 ± 0.20
	axial	3	4.1 ± 0.25	32 ± 5	22.1 ± 1.3
Decellularized	radial	3	1.3 ± 0.20	145 ± 11	2.4 ± 0.20
	axial	3	6.3 ± 0.25	35 ± 5	29.6 ± 1.5

**Table 3:** Effect of the specific surface of PCL, PLGA, and PLCL scaffolds on degradation.

Polymer	PCL			PLGA			PLCL		
Form-factor	Rod	Film	Fibrous material	Rod	Film	Fibrous material	Rod	Film	Fibrous material
Dimensions	⌀ 1.7 mm	Thickness 0.1 mm	⌀ 0.0016 mm	⌀ 2.3 mm	Thickness 0.1 mm	⌀ 0.0049 mm	⌀ 2.5 mm	Thickness 0.2 mm	⌀ 0.0036 mm
Specific surface area, m^2^/g	0.002	0.016	2.1	0.0013	0.016	0.62	0.0012	0.008	0.88
Original M_n_, kDa	96	50	111	63	58	69	91	36.6	54
Crystallinity, %	64	58	35	75:25	75:25	70:30	70:30	74:26	70:30
M_n_ decreasing after 140 days in PBS at 37 °C, %	0	11	36	52	38	15	40	52	14
M_n_ decreasing after 140 days in DMEM at 37 °C, %	-	25	86	94	86	68	89	-	62
Reference	[69]	[101]	-	[101]	[102]	-	[102]	[103]	-

## Data Availability

The data presented in this study are available in Supplementary Materials. Additional data related to this study are available on request from the corresponding author.

## Supplementary Materials

The following supporting information can be downloaded at: https://www.mdpi.com/article/10.3390/biomedicines11030745/s1. Figure S1: The micrographs of nonwoven PCL scaffolds before the destruction test and after 14 days of exposition in test media. Figure S2: The histograms of fiber size distribution calculated with data obtained by the analysis of micrographs of nonwoven PLCL scaffolds before the destruction test and after 14 days of exposition in test media. Figure S3: The histograms of fiber size distribution calculated with data obtained by the analysis of micrographs of nonwoven PLGA scaffolds before the destruction test and after 14 days of exposition in test media. Figure S4: DSC curves of biocompatible scaffolds derived from: A—PLGA; B—PCL; C—PLCL; D—the 1H-NMR spectrum of poly(L-lactide-co-ε-caprolactone) units.
