# Supplementary material for: Biomechanical Behaviors and Degradation Properties of Multilayered Polymer Scaffolds: The Phase Space Method for Bile Duct Design and Bioengineering
Source: Biomedicines. 2023 Mar 1;11(3):745. doi: 10.3390/biomedicines11030745 (PMC10044742; doi:10.3390/biomedicines11030745)
Supplement: Supplementary file 1 [file biomedicines-11-00745-s001.zip › biomedicines-2219519-supplementary.pdf]

Supplementary Figures

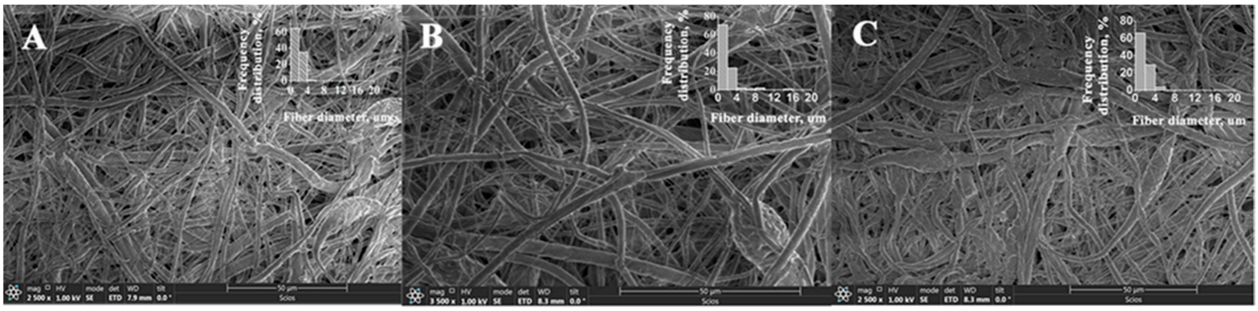

dav=1.6 um; σ =1.0 um   dav =1.5 um; σ =1.87 um   dav =1.5 um; σ =1.2 um

Figure S1. The micrographs of nonwoven PCL scaffolds and the histograms of fiber size distribution before the destruction test (A), and after 14 days of exposition in test media: B – Ox Bile; C - Fenton's reagent. The insert shows the histogram of fiber size distribution. **dav** - average diameter of fiber, µm; σ – standard deviation, µm. Magnification of A, C:×2500; B:×3500.

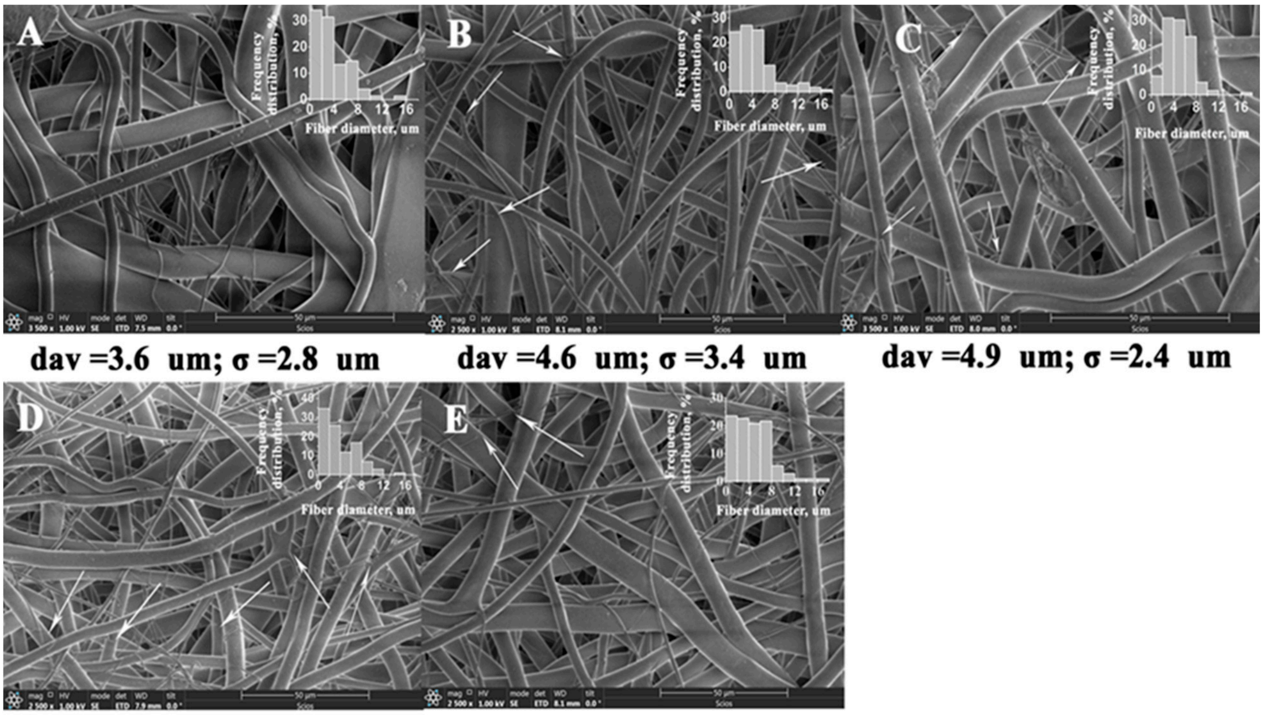

dav =3.6 um; σ =2.8 um   dav =4.6 um; σ =3.4 um   dav =4.9 um; σ =2.4 um

dav =3.8 um; σ =3.2 um   dav =4.7 um; σ =3.1 um

Figure S2. The micrographs of nonwoven PLCL scaffolds and the histograms of fiber size distribution before the destruction test (A), and after 14 days of exposition in test media: B) – Ox Bile; C) – Fenton's reagent; D) – DMEM+FBS; E) – PBS. Magnification of B, D, E: ×2500; A, C: ×3500. **dav** - average diameter of fiber, µm; σ –standard deviation, µm. The arrows indicate the breaking of individual fibers.

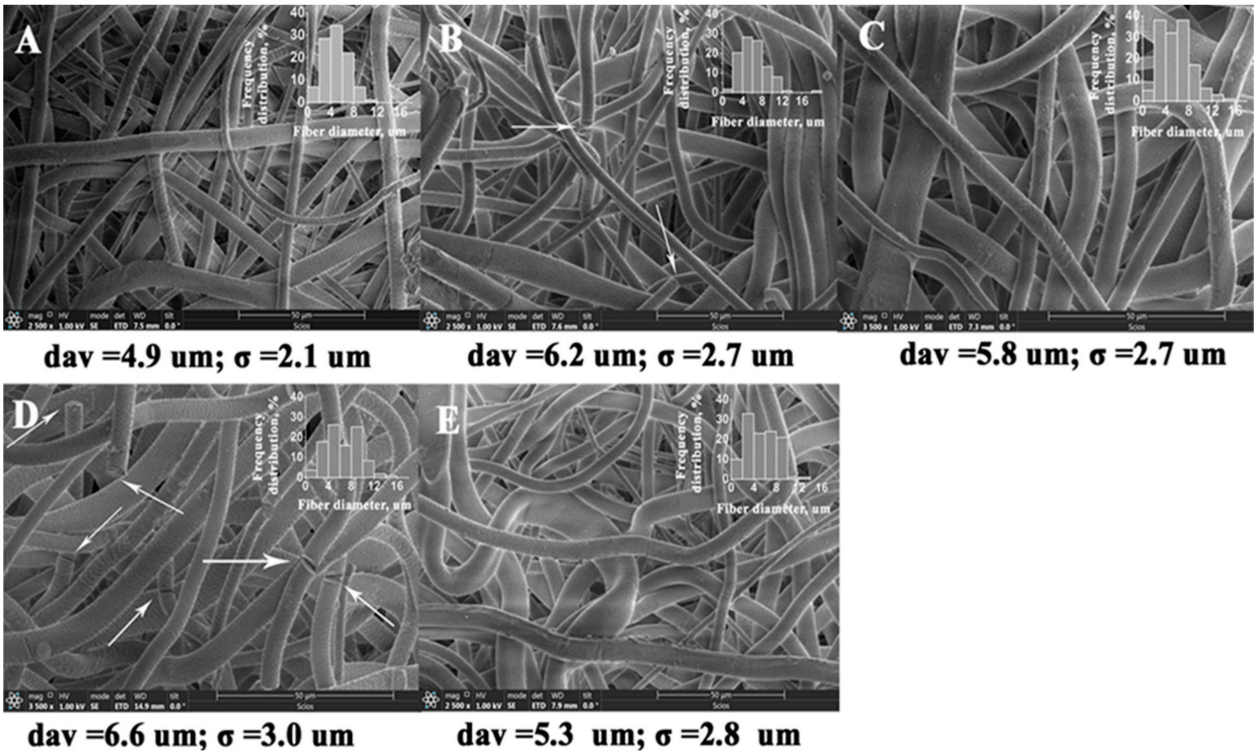

Figure S3. The micrographs of nonwoven PLGA scaffolds and the histograms of fiber size distribution before the destruction test (A), and after 14 days of exposition in test media: B – Fenton's reagent; C - DMEM+FBS; D - Ox Bile; E - Water. Magnification of A, B, E: ×2500; C, D: ×3500. **dav** - average diameter of fiber, µm; σ – standard deviation, µm. The arrows indicate the breaking of individual fibers.

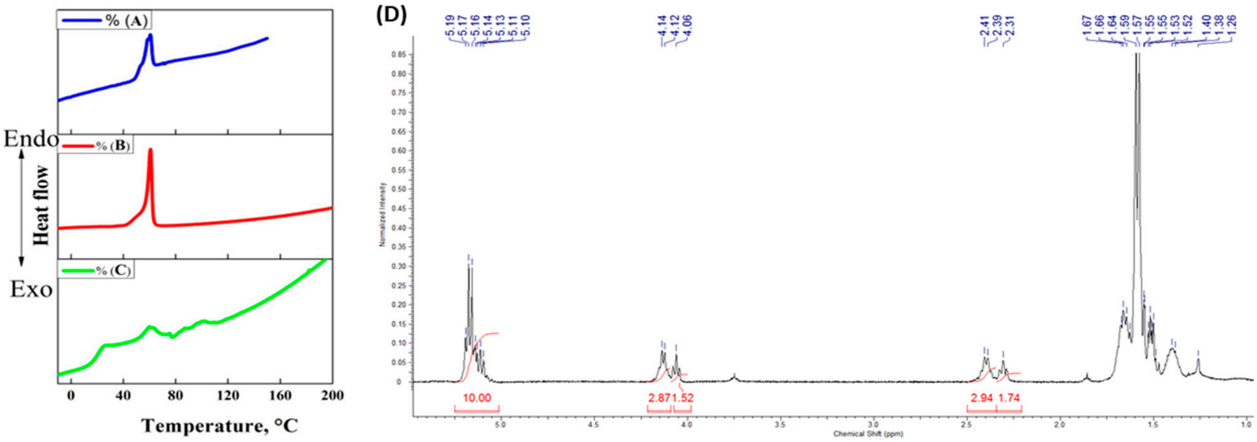

Figure S4. DSC curves of biocompatible scaffolds derived from: A – PLGA; B – PCL; C – PLCL; D – the 1H-NMR spectrum of poly(L-lactide-co-ε-caprolactone) units.
